# Blood Pressure Abnormalities Associated with Gut Microbiota-Derived Short Chain Fatty Acids in Children with Congenital Anomalies of the Kidney and Urinary Tract

**DOI:** 10.3390/jcm8081090

**Published:** 2019-07-24

**Authors:** Chien-Ning Hsu, Pei-Chen Lu, Chih-Yao Hou, You-Lin Tain

**Affiliations:** 1 Department of Pharmacy, Kaohsiung Chang Gung Memorial Hospital and College of Medicine, Chang Gung University, Kaohsiung 833, Taiwan; 2 School of Pharmacy, Kaohsiung Medical University, Kaohsiung 807, Taiwan; 3 Department of Pediatrics, Kaohsiung Chang Gung Memorial Hospital and College of Medicine, Chang Gung University, Kaohsiung 833, Taiwan; 4 Department of Seafood Science, National Kaohsiung University of Science and Technology, Kaohsiung 811, Taiwan; 5 Institute for Translational Research in Biomedicine, Kaohsiung Chang Gung Memorial Hospital and Chang Gung University, College of Medicine, Kaohsiung 833, Taiwan

**Keywords:** ambulatory blood pressure monitoring, butyrate, congenital anomalies of the kidney and urinary tract (CAKUT), cardiovascular disease, children, chronic kidney disease, gut microbiota, hypertension, propionate, short chain fatty acid

## Abstract

Both kidney disease and hypertension can originate from early life. Congenital anomalies of the kidney and urinary tract (CAKUT) are the leading cause of chronic kidney disease (CKD) in children. Since gut microbiota and their metabolite short chain fatty acids (SCFAs) have been linked to CKD and hypertension, we examined whether gut microbial composition and SCFAs are correlated with blood pressure (BP) load and renal outcome in CKD children with CAKUT. We enrolled 78 children with CKD stage G1–G4. Up to 65% of children with CAKUT had BP abnormalities on 24 h ambulatory blood pressure monitoring (ABPM). CKD children with CAKUT had lower risk of developing BP abnormalities and CKD progression than those with non-CAKUT. Reduced plasma level of propionate was found in children with CAKUT, which was related to increased abundance of phylum *Verrucomicrobia*, genus *Akkermansia,* and species *Bifidobacterium bifidum*. CKD children with abnormal ABPM profile had higher plasma levels of propionate and butyrate. Our findings highlight that gut microbiota-derived SCFAs like propionate and butyrate are related to BP abnormalities in children with an early stage of CKD. Early assessments of these microbial markers may aid in developing potential targets for early life intervention for lifelong hypertension prevention in childhood CKD.

## 1. Introduction

Congenital anomalies of the kidney and urinary tract (CAKUT) refer to a various group of structural malformations that are characterized by defects in fetal kidney development [1]. CAKUT account for almost 30 percent of all anomalies identified in the prenatal period [2]. The causes of chronic kidney disease (CKD) in childhood differs from those in adults, as the largest category among children and adolescents are CAKUT [3]. CAKUT are the leading cause of end stage renal disease (ESRD) in the pediatric population [4,5]. Importantly, evidence is accumulating that many patients with CAKUT may progress to ESRD during adulthood [6].

Hypertension is the most commonly complication in pediatric CKD. We, and others, have shown that even in mild to moderate CKD, approximately 50% of children have blood pressure (BP) abnormalities [7,8]. Hypertension is associated with a high risk of developing cardiovascular disease (CVD) and more rapid progression of CKD [9,10]. Unlike adults, overt CVD hardly presents in children. Thus, surrogate markers, such as noninvasive measurement of blood pressure (BP) load, arterial stiffness, and vascular phenotype [11], are essential to manage and stratify risk for CVD in pediatric CKD. CKD and hypertension can both originate in early life [12,13]. Prematurity and low birth weight have relatively increased risk for the development of CAKUT, CKD, as well as hypertension later in life [13,14,15]. In view of this, a better understanding of the factors linking CAKUT to hypertension in early stage of CKD is essential to developing potential interventions to halt the growing epidemic of CKD-related diseases.

Recent studies suggest the pathogenic association between gut microbiota and CKD [16,17]. CKD can affect the microbial composition and their metabolites like short chain fatty acids (SCFAs) [18], whereas gut dysbiosis in CKD patients may increase gut microbiota-derived uremic toxins that in turn contribute to CKD progression. Although some microbial markers have been linked to hypertension [19,20,21], the impact of gut microbiota on BP and its association with CAKUT in children with early CKD remains largely unclear.

Given the importance of gut microbiota and their metabolites as risk factors for BP abnormalities and CVD, we analyzed data from the pediatric CKD cohort study to investigate their impact on microbial markers for CVD in children with the focus on CAKUT.

## 2. Materials and Methods

### 2.1. Study Population

From December 2016 to April 2019, we enrolled a total of 125 children and adolescents aged 3 to 18 years with CKD attending the pediatric clinic at Kaohsiung Chang Gung Memorial Hospital, a medical center in Taiwan. This prospective cohort study was approved by the Institution Review Board and Ethics Committee of Chang Gung Medical Foundation, Taoyuan, Taiwan (Permit number: 201601181A3). Our study protocol was adherent to the principle of the 1964 Declaration of Helsinki and its later amendments. Written informed consent was obtained from all participants. CKD is defined as decreased kidney function or presence of persistent kidney damage over at least three months [22]. Renal function was determined by estimated glomerular filtration rate (eGFR) using the Schwartz formula according to body height and serum creatinine (Cr) level [23]. Kidney damage refers to structural abnormalities or functional abnormalities, whether established via renal biopsy or imaging studies, or inferred from markers such as urinary sediment abnormalities or proteinuria. Patients were excluded, if they (1) were already documented as pregnant; (2) had a history of congenital heart disease; (3) had an eGFR <15 mL/min/1.73 m^2^, were on dialysis maintenance, or had ever received renal transplantation; (4) were unable to complete follow-up protocol or cooperate with assessment. All participants were categorized into eGFR category G1 (eGFR ≥90 mL/min/1.73 m^2^), G2 (eGFR 60–89 mL/min/1.73 m^2^), G3 (eGFR 30–59 mL/min/1.73 m^2^), or G4 (eGFR 15–29 mL/min/1.73 m^2^). All recruited patients were followed-up every 6 months up to progression to ESRD. Analysis was restricted to children with a baseline eGFR >15 mL/min/1.73 m^2^, measured cardiovascular surrogate markers and fecal microbiota are described in the following section. In the current study, we enrolled a total of 78 children and adolescents with CKD stage G1 to G4 with a 1 year follow-up to calculate the change of eGFR to determine CKD progression. The causes of kidney diseases were divided into two categories: CAKUT or non-CAKUT. CAKUT structural anomalies range from renal agenesis, kidney hypo-/dysplasia, horseshoe kidney, duplex collecting system, multi-cystic kidney dysplasia, posterior urethral valves, and ureter abnormalities [24].

### 2.2. Biochemical Analysis

Fasting plasma specimens, spot urine, and fecal samples were aliquoted and stored at −8 °C until analysis. Blood urea nitrogen (BUN), creatinine, uric acid, glucose, total cholesterol, low-density lipoprotein (LDL), triglyceride, sodium, potassium, calcium, phosphate, hemoglobin, hematocrit, and urine total protein-to-creatinine ratio were measured by the hospital’s central laboratory as described previously [8].

### 2.3. Office Blood Pressure and 24 h Ambulatory Blood Pressure Monitoring (ABPM)

Office BP measurements were taken at a clinic visit after 5 min sitting at rest with at least 1 min between recordings. The mean value was used as the participant’s office BP for analysis. We used the Oscar II monitoring device (SunTech Medical, Morrisville, NC, USA) to measure BP and pulse rate at 20 min intervals over 24 h. The 24 h ambulatory blood pressure monitoring (ABPM) data were collected for subjects aged 6–18 years, handled by an experienced specialist nurse as described previously [25]. The participants and their parents were asked to keep a diary of sleeping and waking times, as well as activities that may influence BP measurements, including exercise and stressful situations. Only measurements with a systolic BP of 50–200 mm Hg, a diastolic BP of 30–100 mm Hg, and a heart rate of 30–200 beats per minute were accepted as valid and included in analysis. An abnormal ABPM profile was determined based on (1) awake, asleep, systolic, or diastolic BP loads exceeding the 95th percentile based on gender and height using ABPM reference data [26]; (2) awake, asleep, systolic, or diastolic BP load of 25% or greater; and (3) asleep decrease of BP load by less than 10% compared with average awake BP load. Next, the ambulatory arterial stiffness index (AASI) is an index derived from 24 h ABPM for the evaluation of arterial stiffness [27]. The diastolic BP was plotted against systolic BP using the individual 24 h ABPM readings to calculate the linear regression slope. The AASI was defined as one minus the regression slope [27].

### 2.4. Gas Chromatography-Flame Ionization Detector (GC-FID)

Plasma acetate, butyrate, and propionate levels were measured using gas chromatography-mass spectrometry (GCMS-QP2010; Shimadzu, Kyoto, Japan) with flame ionization detector (FID), as previously reported [28]. Separation was performed on the SGE BP GC column (21 × 0.5 µm, 30 m × 0.53 mm; Shimadzu GLC Ltd., Tokyo, Japan). The working solutions of acetate, butyrate, and propionate used as internal and external standards were at the concentration of 10 mM and kept at −20 °C in the freezer. Dry air, nitrogen, and hydrogen were supplied to the FID at 300, 20, and 30 mL/min, respectively. An aliquot of 2 µl sample was injected into the column. The inlet and FID temperature were set at 200 °C and 240 °C, respectively. The total running time was 17.5 min. Analytical standard grades used as internal standards for acetate and propionate were obtained from Sigma-Aldrich (St. Louis, MO, USA) and for butyrate was from Chem Service (West Chester, PA, USA).

### 2.5. Analysis of Gut Microbiota Composition

As described previously [29], Metagenomic DNA was extracted from frozen fecal samples after centrifugation. According to the manufacturer’s protocol, all polymerase chain reaction amplicons were mixed together and sent to the Biotools Co., Ltd. (Taipei, Taiwan) for sequencing using Illumina Miseq platform (Illumina, San Diego, CA, USA). The sequences were analyzed using QIIME version 1.9.1. Sequences (Illumina, San Diego, CA, USA) with a distance-based similarity of 97% or greater were grouped into operational taxonomic units (OTUs) using the USEARCH algorithm. The phylogenetic relationships were determined based on a representative sequence alignment using Fast-Tree. Shannon’s index accounting for both abundance and evenness of the taxa present was analyzed by QIIME version 1.9.1. We evaluated the β-diversity changes in gut microbiota across groups by the Partial Least Squares Discriminant Analysis (PLS-DA) and the Analysis of Similarities (ANOSIM). To determine the significantly differential taxa, we applied linear discriminant analysis effect size (LEfSe) to compare samples between groups. The LEfSe uses linear discriminant analysis (LDA) to estimate the effect size of each differentially abundant feature [30]. The threshold of the linear discriminant was set to three.

### 2.6. Statistical Analysis

Data were expressed as medians (25th, 75th percentile) for continuous variables while categorical variables were expressed as number (%). The Mann–Whitney U-test or Chi-square test was used to test the differences in variables between children with CAKUT and non-CAKUT. The associations between variables were examined using Pearson’s correlation coefficient. A value of *p* < 0.05 was considered statistically significant. Analyses were performed using the Statistical Package for the Social Sciences (SPSS) software 14.0 (SPSS Inc., Chicago, IL, USA).

## 3. Results

There was a total of 78 children and adolescents with CKD in this study, including 51 G1 subjects (65.4%), 17 G2 subjects (21.8%), 16 G3 subjects (20.5%), and one G4 subject (1.3%). Our study population was slightly predominant male (M:F = 1.7:1). The median age was 11.2 (7.4–15.2) years. The median eGFR was 100.3 (81.5–115.7) mL/min/1.73 m^2^, indicating most participants were in an early stage of CKD. As shown in Table 1, CAKUT account for approximately 70 percent (57/78) of patients. The rate of CKD progression in 1 year follow-up was 15.8% (9/57) in the CAKUT group, a lesser extent than the non-CAKUT group which had a rate of 33.3% (7/21). In the CAKUT group, systolic and diastolic blood pressures, body mass index, and plasma levels of creatinine and uric acid were lower in the children compared to those in adolescents in an age-dependent manner. However, CAKUT adolescents had a lower eGFR but a higher rate of CKD progression compared to children. Unlike the CAKUT group, systolic and diastolic blood pressures and body mass index were not significantly different between children and adolescents in the non-CAKUT group. Additionally, we observed that the CAKUT group had a lower rate of hypertension, lower eGFR, lower urine total protein-to-creatinine ratio, lower plasma levels of LDL and triglyceride, but higher plasma levels of calcium and phosphate compared to those with non-CAKUT. While in adolescents, most parameters were not different between the CAKUT and non-CAKUT group.

Among them, 29 cases (37.2%) had office BP exceeding the 95th percentile for age, gender, and height. A total of 55 patients (70.5%) aged 6–18 years had undergone 24 h ABPM and 69% (38/55) of them had at least one BP load abnormality (Table 2). Among them, 12 cases (31.6%) experienced CKD progression. Conversely, none of the cases with normal ABPM developed CKD progression (*p* = 0.009). The ABPM identified 14 (25%), 16 (29%), and 15 participants (27%) with systolic BP or diastolic BP load >95th percentile at 24 h, awake, and asleep stages, respectively. Other ABPM abnormalities included 30 patients (55%) with BP load ≥25% and 29 patients (53%) with non-dipping nocturnal SBP. The cases with SBP or DBP load >5th percentile at nighttime and BP load ≥25% were lesser in the CAKUT vs. the non-CAKUT group. The AASI, an index of arterial stiffness, was not different between the CAKUT and non-CAKUT group.

We next analyzed plasma SCFAs levels. As shown in Table 3, children with CAKUT had lower plasma levels of propionate compared to those with non-CAKUT. However, plasma levels of acetate and butyrate were not different between the two groups. Using data pooled from all subjects, correlations between plasma SCFAs levels and biochemical data, BP load, and AASI were analyzed. We observed that plasma acetate level was positively correlated with BUN (r = 0.289, *p* = 0.011), total cholesterol (r = 0.278, *p* = 0.015), and LDL (r = 0.285, *p* = 0.013). Plasma propionate level was positively correlated with SBP (r = 0.288, *p* = 0.012), eGFR (r = 0.295, *p* = 0.01), urinary total protein-to-creatinine ratio (r = 0.483, *p* < 0.001), total cholesterol (r = 0.529, *p* < 0.001), LDL (r = 0.423, *p* < 0.001), and uric acid (r = 0.282, *p* = 0.014).

We found that plasma propionate level was significantly higher in children with abnormal 24 h, daytime, and nighttime BP, and BP load than those with normal profile (Table 4). Additionally, CKD children with abnormal 24 h, daytime, and nighttime BP in ABPM profile had a higher plasma butyrate level compared to those with a normal ABPM profile.

**Figure 1:**
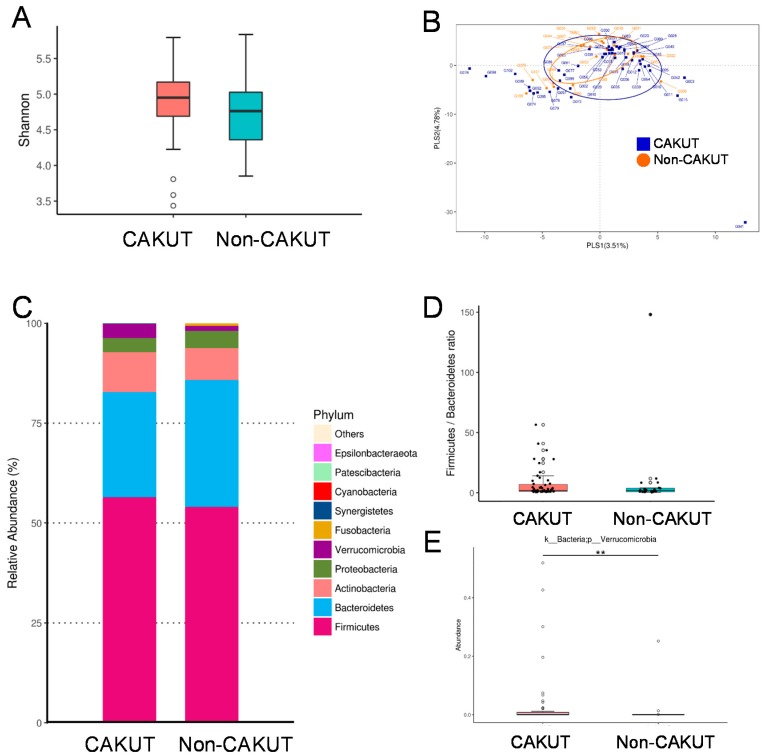
(**A**) Variation in fecal bacterial α-diversity analyzed by the Shannon’s diversity indexes. (**B**) β-diversity changes in gut microbiota across groups by the Partial Least Squares Discriminant Analysis (PLS-DA). (**C**) Relative abundance of the top 10 phylum of the gut microbiota between the CAKUT and non-CAKUT group. (**D**) Ratio of *Firmicutes* to *Bacteroidetes* (F/B), as a marker of gut dysbiosis. (**E**) The abundance of phylum *Verrucomicrobia* in CKD children with CAKUT vs. non-CAKUT. The double asterisk indicates *p* < 0.01.

We further analyzed gut microbiota composition of children and adolescents with CKD. The Shannon index, an index of α-diversity, was analyzed to determine species richness and it was found that there was no significant difference between the CAKUT and non-CAKUT group (Figure 1A; *p* = 0.101). The β-diversity analysis indicates the extent of similarity between microbial communities. The score plots of PLS-DA analysis showed that two groups were not well-separated (Figure 1B) and the ANOSIM analysis between the CAKUT and non-CAKUT group did not reach significance (*p* = 0.069). We observed that the main phyla were *Firmicutes, Bacteroidetes, Actinobacteria*, *Proteobacteria,* and *Verrucomicrobia* (Figure 1C). At the phylum level, the abundance of *Verrucomicrobia* was higher in the CAKUT vs. the non-CAKUT group (*p* < 0.01). The *Firmicutes* to *Bacteroidetes* ratio, a microbial biomarker for hypertension [19], was not different between children with CAKUT and those with non-CAKUT (Figure 1D).

**Figure 2:**
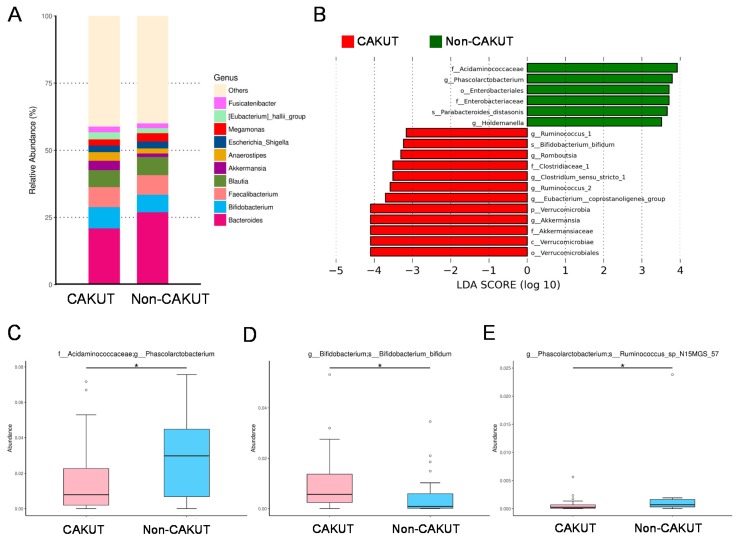
(**A**) Relative abundance of the top 10 genera of the gut microbiota between the CAKUT and non-CAKUT group. (**B**) Linear discriminant analysis effect size (LEfSe) to identify the taxa that were significantly different between the CAKUT and non-CAKUT group. The threshold of the linear discriminant was set to 3. (**C**) The abundance of genus *Phascolarctobacterium*, (**D**) species *Bifidobacterium bifidum*, and (**E**) species *Ruminococcus_sp_N15MGS_57* in CKD children with CAKUT vs. non-CAKUT. The asterisk indicates *p* < 0.05.

At the genus level (Figure 2A), the abundances of the top 10 genera were not different between the CAKUT and non-CAKUT group. Additionally, we performed the LEfSe algorithm to identify metagenomic biomarkers (Figure 2B). Our results identified genera *Akkermansia, Eubacterium, Ruminococcus, Clostridium,* and *Romboutsia* in the CAKUT group are detected by LEfSe with a high LDA score (more than three orders of magnitude), reflecting marked abundance in CAKUT and low abundance in the non-CAKUT group. Children with CAKUT had decreased abundance of genus *Phascolarctobacterium* (*p* = 0.044) (Figure 2C). Additionally, the CAKUT group showed increased species *Bifidobacterium bifidum* (*p* = 0.012) (Figure 2D), but decreased abundance of species *Ruminococcus_sp_N15MGS_57* (*p* = 0.016) (Figure 2E).

## 4. Discussion

Our study describes, for the first time, that gut microbiota-derived SCFAs link BP abnormalities to CAKUT in children and adolescents with an early stage of CKD. The key findings can be summarized as follows: (1) Up to 69% of children and adolescents with CKD stage G1–G4 had BP abnormalities in ABPM; (2) The rate of CKD progression in the 1 year follow-up was 15.8% and 33.3% in the CAKUT and non-CAKUT group, respectively; (3) Within the CAKUT group, adolescents had a lower eGFR and higher rate of CKD progression compared to children; (4) CKD children with CAKUT had lower risk of developing nighttime hypertension and BP load ≥25% than the non-CAKUT group; (5) Children with CAKUT had lower plasma levels of propionate compared to those with non-CAKUT; (6) CKD children with an abnormal ABPM profile had higher plasma levels of propionate and butyrate; and (7) CKD children with CAKUT had a higher abundance of phylum *Verrucomicrobia*, genus *Akkermansia,* and species *Bifidobacterium bifidum* than those with non-CAKUT.

In the current study, approximately 70% of CKD children had BP abnormalities in ABPM, while those with CAKUT displayed BP abnormalities to a lesser extent. This result ties in well with previous studies wherein hypertension is highly prevalent in CKD children, even in an early stage of CKD [8,9,10,31]. The present study also confirmed the notion that ABPM is superior to office BP in identifying children with BP abnormalities [11]. Children with CAKUT are expected to progress to ESRD because the congenital reduction in nephron number eventually overloads the remaining nephrons [1]. In the ItalKid Study, in a population-based registry of children with CAKUT, the risk of progressing to ESRD by the age of 20 was 68% [32]. In our overall study population, the 1 year CKD progression rate was 20.5%, which is comparable to that reported previously [33,34]. Of note is that children with CAKUT had a lower CKD progression rate compared to those in the non-CAKUT group. Our data supports the notion that many children with mild to moderate renal hypoplasia/dysplasia can maintain stable CKD during childhood and progress slowly to ESRD until adulthood [6]. Additionally, our data showed that children with an abnormal ABPM profile are prone to experience CKD progression. Hypertension is a risk factor for CKD progression [33], so it is not surprising to see CAKUT associated with low occurrence of BP abnormalities and CKD progression. Consistent with a previous study showing that patients with CAKUT survived longer than non-CAKUT controls due to lower cardiovascular mortality [5,6], our results showed the proportion of BP abnormalities and CKD progression was lower in children with CAKUT than in those with non-CAKUT. We previously showed that AASI, an index of arterial stiffness, was correlated with BP abnormalities in children with an early stage of CKD [25]. Nevertheless, we found no difference in AASI between CAKUT and non-CAKUT.

According to our data, children with CAKUT had increased abundance of phylum *Verrucomicrobia*, genera *Akkermansia, Ruminococcus*, *Clostridium*, and *Romboutsia*, and species *Bifidobacterium bifidum*. *Akkermansia*, a genus in the phylum *Verrucomicrobia*, is known as a beneficial gut microbe [35]. Similarly, *Bifidobacterium bifidum* has been shown to benefit cardiovascular health [36]. A previous study reported that the abundance of genera *Akkermansia, Ruminococcus, Clostridium,* and *Roseburia* were deficient in mice with hypertension [37]. Overall these observations suggest that these certain bacteria populations might have beneficial properties in early-stage CAKUT children to halt the progression of hypertension and CKD. The *Firmicutes* to *Bacteroidetes* ratio has been used as a microbial marker for hypertension [19,20]. However, we did not find a difference in the *Firmicutes* to *Bacteroidetes* ratio between CAKUT and non-CAKUT, regardless of non-CAKUT being associated with increased occurrence of BP abnormalities. This is possibly because we analyzed CKD children preceding hypertension onset but not in the stage of established hypertension. Another possible reason for not reaching significance may be due to a small sample size with insufficient power.

Emerging evidence support that SCFAs link gut microbiota to BP regulation [17,18,19,20,21]. From our results it is clear that elevated plasma propionate level is relevant to children with non-CAKUT as well as an abnormal ABPM profile. Intriguingly, SCFAs are generally known to induce vasorelaxation [37]. Accordingly, elevated propionate level is presumed to reduce rather than induce BP. However, a previous study demonstrated that propionate can modify renin release and increase BP in an olfactory receptor 78 (Olfr78)-dependent manner [38]. Additionally, elevated propionate level is related to hypertension in a salt-sensitive hypertension rat model [39]. Our data showed propionate level was positively correlated with several risk factors related to hypertension, such as urinary total protein-to-creatinine ratio, total cholesterol, LDL, and uric acid. Thus, whether propionate plays a beneficial or detrimental role in the development of hypertension in children with an early stage of CKD deserves further clarification. The succinate pathway is the dominant route for the generation of propionate, which is found mainly in *Phascolarctobacterium* spp. [40]. Since the abundance of genus *Phascolarctobacterium* was higher in the non-CAKUT vs. CAKUT group, and non-CAKUT had higher propionate levels than that in CAKUT, whether targeting of genus *Phascolarctobacterium* to lower propionate level may aid in protecting non-CAKUT children against hypertension awaits further elucidation.

Although plasma butyrate level was not different between the CAKUT and non-CAKUT group, its level is related to high BP load. The major genera of butyrate-producing microbes include *Coprococcus, Facecalibacterium, Eubacterium*, and *Roseburia* [41]. Our results demonstrated that these butyrate-producing microbes were not different between the CAKUT and non-CAKUT group. Like propionate, butyrate has been reported to display a vasodilatory property [37]. However, the role of butyrate in hypertension remains controversial. Although low abundance of butyrate-producing microbes and blood butyrate level were found in spontaneously hypertensive rats [19,42], butyrate like propionate is a ligand for Olfr78 that can induce renin release and elevate BP [38]. Additional studies are required to clarify whether these alterations of gut microbiota-derived SCFAs are involved in the development of hypertension and CKD progression in patients with early stage of CKD. Emerging evidence suggests several mechanisms by which intestinal dysbiosis associated with CKD contributes to cardiovascular disease, such as defects in intestinal barrier function, inflammation, and reduced clearance of microbiota-derived uremic toxins [16,43]. Conversely, microbiota-targeted interventions have been shown to improve cardiovascular outcomes in CKD [18,44]. Despite recent advances in exploring molecular mechanisms of CAKUT [1,24], the pathogenic link between gut dysbiosis and cardiovascular morbidity in CKD children with CAKUT remains largely unknown. In the present study, we see great opportunities for the potential use of gut microbiota and its derived SCFAs as markers for BP abnormalities in children with CAKUT. The possibility of microbiota-targeted interventions in preventing hypertension and cardiovascular mobility in CKD youths with CAKUT warrants further investigation.

Our study has several limitations. First, the 1 year follow-up period is short. More CV assessments with longer follow-ups are required in view of the long-term nature of childhood CKD. Second, we acknowledge that our sample of CKD children from one hospital may not be representative of the population as a whole. Larger numbers of patients recruited via multi-center cohorts may be warranted in the future to elucidate the true relationship. Third, we found a correlation between certain taxa and BP abnormalities but we do not reveal the pathophysiological mechanism by which those specific taxa contribute to the development of hypertension. Additionally, the composition of gut microbiota can change in an age-dependent manner. Future studies should aim to replicate results in a larger cohort comparing CAKUT with non-CAKUT that are matched by age. Last, we used ABPM reference values from studies performed in Germany [26]. Ethnic differences should be considered.

## 5. Conclusions

In conclusion, BP abnormalities are highly prevalent in CAKUT, the most important cause of CKD in children. Our results cast a new light on the link between gut microbiota, SCFAs, BP load, and CKD progression in youths with early stage of CKD. Since both kidney disease and hypertension can originate in earliest childhood, early detection of microbial markers related to BP abnormalities and CKD progression may aid in improving cardiovascular outcome in children with CAKUT.

## Figures and Tables

**Table 1:** Clinical, anthropometric and biomedical characteristics in children and adolescents with chronic kidney disease (CKD).

Group	CAKUT		Non-CAKUT	
	N = 57		N = 21	
	Children	Adolescents	Children	Adolescents
	N = 39	N = 18	N = 6	N = 15
Gender: M:F	24:15	11:7	5:1	9:6
CKD staging				
Stage G1	33	6	5	7
Stage G2	3	9	0	5
Stage G3	3	2	1	3
Stage G4	0	1	0	0
Age, years	7.5 (5.5–10.3)	15.8 (14.1–17.5) ^a^	10.2 (7.9–11.4)	15.7 (13.9–16.9) ^a^
Body height, percentile	50 (15–75)	50 (25–77.5)	25 (3–58.8)	25 (3–50)
Body weight, percentile	25 (15–75)	50 (12–85)	25 (3–85)	25 (15–85)
Systolic blood pressure, mmHg	105 (101–113)	121 (111–130) ^a^	114 (105–119)	124 (112–136)
Diastolic blood pressure, mmHg	69 (64–73)	74 (71–82) ^a^	75 (63–82)	70 (64–78)
Body mass index, kg·m^−2^	15.6 (14.7–17.7)	20.8 (17.8–24.8) ^a^	18.3 (13.9–23.2)	20.3 (18.3–23.6)
Hypertension (by office BP)	12 (30%)	6 (33%)	5 (83%) ^b^	6 (40%)
Blood urea nitrogen, mg/dL	13 (10–16)	13 (12–15)	14 (11–20)	12 (11–15)
Creatinine, mg/dL	0.5 (0.42–0.56)	0.86 (0.64–0.97) ^a^	0.37 (0.19–0.75)	0.73 (0.58–1) ^a^
eGFR, mL/min/1.73 m^2^	104 (96–116)	82 (71–101) ^a^	136 (103–393) ^b^	88 (71–113) ^a^
CKD progression	2 (5.1%)	7 (38.9%) ^a^	1 (16.7%)	5 (33.3%)
Urine total protein-to-creatinine ratio, mg/g	49 (34–72)	71 (29–172)	2364 (709–12,293) ^b^	273 (51–1282)
Hemoglobin, g/dL	13.4 (12.8–14)	14.2 (12.2–16.2)	13.4 (12.7–14.5)	12.9 (11.9–14.2)
Hematocrit, %	39.4 (37.9–41.3)	42.2 (37.1–46.1)	38.8 (37.2–40.3)	38.1 (36–41.6)
Total cholesterol, mg/dL	174 (155–194)	146 (130–166) ^a^	239 (158–353)	185 (132–207)
LDL, mg/dL	89 (74–110)	80 (59–99)	140 (92–252) ^b^	96 (69–129)
Triglyceride, mg/dL	61 (43–87)	65 (51–106)	98 (91–159) ^b^	66 (45–156)
Uric acid, mg/dL	4.8 (3.9–5.4)	5.9 (5.5–6.8) ^a^	7.6 (4.1–8.7)	6.9 (5.2–9)
Sodium, mEq/L	141 (139–142)	142 (140–143)	141 (137–142)	140 (140–142)
Potassium, mEq/L	4.4 (4.3–4.7)	4.4 (4.2–4.6)	4.2 (3.6–4.9)	4.4 (4–4.7)
Calcium, mg/dL	9.9 (9.7–10.1)	9.9 (9.5–10.2)	8.5 (7.3–9.8) ^b^	9.6 (9.2–9.6)
Phosphate, mg/dL	5 (4.7–5.3)	4.5 (4.1–4.9) ^a^	4.5 (4.1–4.7) ^b^	4.3 (3.9–4.4)

Data are medians (25th, 75th percentile) or n (%). ^a^ *p* < 0.05 children vs. adolescent by the Chi-square test or Mann–Whitney U-test. ^b^ *p* < 0.05 CAKUT vs. non-CAKUT by the Chi-square test or Mann–Whitney U-test. Children = up to 12 years of age. Adolescents = aged 13–18 years. BP = blood pressure; CAKUT = Congenital anomalies of the kidney and urinary tract; eGFR = estimated glomerular filtration rate; LDL = low-density lipoprotein.

**Table 2:** ABPM profile and arterial stiffness assessment in children with CKD.

Group	CAKUT	Non-CAKUT
24 h ABPM	N = 37	N = 18
Abnormal ABPM profile (with any of the following abnormalities)	24 (65%)	14 (78%)
Average 24 h BP >95th percentile	7 (19%)	7 (39%)
Average daytime BP >95th percentile	8 (22%)	8 (44%)
Average nighttime >95th percentile	7 (19%)	8 (44%) *
BP load ≥25%	16 (43%)	14 (78%) *
Nocturnal decrease of BP <10%	20 (54%)	9 (50%)
Arterial stiffness assessment		
AASI	0.35 (0.22–0.46)	0.38 (0.25–0.49)

Data are medians (25th, 75th percentile) or n (%). * *p* < 0.05 by the Chi-square test or Mann–Whitney U-test. ABPM = ambulatory blood pressure monitoring. AASI = ambulatory arterial stiffness index.

**Table 3:** Plasma levels of acetate, propionate, and butyrate in children with CKD.

Plasma Level	CAKUT	Non-CAKUT
Acetate, μM	56.4 (48.5–68.1)	57.9 (46–83.4)
Propionate, μM	1.8 (1.5–2.2)	2.5 (1.6–3.3) *
Butyrate, μM	1.4 (1.1–1.7)	1.8 (1.2–2.2)

Data are medians (25th, 75th percentile) or n (%). * *p* < 0.05 by the Mann–Whitney U-test.

**Table 4:** Plasma short chain fatty acid (SCFA) levels vs. BP and ABPM profile in children with CKD.

BP	n	Acetate	Propionate	Butyrate
24 h BP				
Abnormal	14	65.7 (49.4–73.5)	2.4 (1.6–3.4) *	1.9 (1.2–2.6) *
Normal	40	56.1 (46–66.6)	1.9 (1.5–2.2)	1.5 (1.1–1.6)
Daytime BP				
Abnormal	16	65.7 (47.3–77.2)	2.4 (1.6–3.3) *	1.8 (1.2–2.4) *
Normal	38	56.1 (45.5–65.7)	1.9 (1.5–2.2)	1.4 (1.1–1.6)
Nighttime BP				
Abnormal	15	68.1 (50.5–79.1)	2.5 (1.6–3.5) *	1.9 (1.2–2.5) *
Normal	39	55.8 (45.9–65.3)	1.8 (1.5–2.2)	1.4 (1.1–1.6)
BP load				
Abnormal	30	59.5 (46.1–73.5)	2.2 (1.6–2.8) *	1.4 (1.1–1.9)
Normal	24	52.5 (46.2–65.2)	1.8 (1.4–2.1)	1.5 (1.2–1.6)
Night dipping				
Abnormal	29	58.9 (48–75.3)	2 (1.6–2.6)	1.3 (0.8–1.7)
Normal	25	55.8 (44.5–66.2)	1.8 (1.5–2.4)	1.5 (1.3–1.9)
ABPM profile				
Abnormal	38	59 (46.4–68.6)	2 (1.7–2.7)	1.4 (1–1.8)
Normal	16	54.2 (42.7–64.5)	1.8 (1.3–2.2)	1.5 (1.3–1.8)
Office BP				
Abnormal	29	54.3 (49.4–68.6)	2 (1.7–2.7)	1.5 (1.2–1.9)
Normal	47	56.9 (46.5–71.5)	1.8 (1.5–2.2)	1.4 (1–1.7)

Data are medians (25th, 75th percentile) or n (%). * *p* < 0.05 by the Mann–Whitney U-test.
